# Predictors of Organisational Commitment and Retention Intent Among Midcareer Nurses: Addressing the Midcareer Dip in Taiwan

**DOI:** 10.1155/jonm/2659894

**Published:** 2026-04-09

**Authors:** Ching-Wen Wei, Yen-Chin Chen, Ching-Wen Hu

**Affiliations:** ^1^ Institute of Clinical Nursing, National Chung Hsing University, Taichung, Taiwan, nchu.edu.tw; ^2^ Department of Nursing, College of Medicine, National Sun Yat-sen University, Kaohsiung, Taiwan, nsysu.edu.tw; ^3^ School of Nursing, College of Nursing, Kaohsiung Medical University, Kaohsiung, Taiwan, kmu.edu.tw; ^4^ Department of Nursing, Tungs’ Taichung MetroHarbor Hospital, Taichung, Taiwan, sltung.com.tw

**Keywords:** health workforce, midcareer nurses, organisational commitment, workload, work perceptions

## Abstract

**Background:**

The global nursing workforce shortage has been intensified by the COVID‐19 pandemic, placing frontline nurses under sustained pressure and potentially diminishing their organisational commitment. This study aimed to examine differences in organisational commitment among midcareer nurses in Taiwan across demographic and work‐related characteristics and to investigate the relationships between work excitement, work frustration and organisational commitment in order to identify key predictors of organisational commitment.

**Methods:**

A cross‐sectional design with convenience sampling was employed. A total of 240 registered female nurses working at or above the regional hospital level in northern Taiwan were recruited between July and December 2024 through online professional nursing networks. Data were collected using a demographic questionnaire, the Work Excitement Scale, the Work Frustration Scale and the Organisational Commitment Scale. Descriptive statistics, independent *t*‐tests, one‐way ANOVA and multiple regression analyses were performed.

**Results:**

Participants had a mean age of 32.54 years and an average of 10.36 years of nursing experience. Higher organisational commitment was observed among nurses who were older, held managerial positions, worked regular day shifts and perceived lighter workloads. Work excitement was positively associated with organisational commitment, whereas work frustration and perceived workload were negative predictors. Importantly, nurses in the midcareer stage (3–8 years of tenure) demonstrated significantly lower organisational commitment compared with those at earlier or later career stages, whereas chronological age was not an independent predictor after adjustment for tenure and other covariates.

**Conclusion:**

Midcareer nurses encounter distinct challenges, including heavy workloads, constrained career advancement and discrepancies between expectations and workplace realities, which together undermine their organisational commitment. As this group represents the core of the nursing workforce, tailored retention strategies are essential. Drawing on international best practices, Taiwan should establish structured professional development and continuing education frameworks that support midcareer nurses across clinical, educational, managerial and research trajectories. Such initiatives are crucial to enhancing job satisfaction, reinforcing commitment and promoting long‐term workforce stability.

## 1. Introduction

In clinical settings, midcareer nurses play a critical role in care delivery through clinical decision‐making and mentoring newly graduated nurses. Prior research has operationalised midcareer nurses using varying criteria, including age thresholds (e.g., 30 years and above) or accumulated clinical experience, to represent a stage of professional consolidation and increased organisational responsibility [1]. At this career stage, nurses face persistent challenges across intrapersonal, interpersonal, organisational and societal levels, which may compromise their well‐being, family roles and career development [2, 3]. These structural and organisational barriers uniquely shape midcareer nurses’ retention and long‐term organisational commitment [4]. However, career progression does not follow a uniform trajectory and may differ across healthcare systems and institutional contexts. Accordingly, in the present study, midcareer nurses are conceptualised as a career‐stage construct rather than a strictly age‐ or tenure‐based classification, with emphasis on work‐related experiences characteristic of the midcareer phase.

Nursing, rooted in service and compassion, remains a profession defined by both dedication and demanding responsibility. Across the globe, the shortage of qualified nurses has persisted as a major workforce challenge, resisting decades of policy efforts. The issue becomes particularly acute in times of large‐scale crises, when workforce volatility rises sharply. The 2019 emergence of a novel coronavirus triggered worldwide disruption and immense loss of life. Nurses, positioned at the frontline of care and prevention, bore a disproportionate share of the physical and emotional strain. Their experiences during this crisis reshaped how many perceived their professional identity and value. Although the postpandemic transition began in 2023 [5], continued viral evolution, workforce depletion and infection risks have prolonged the pressure on those providing direct care. These sustained challenges have been associated with heightened job stress, diminished organisational commitment and increased intentions to leave the profession [6, 7].

The International Council of Nurses reported that by 2021, 62% of member nations faced heavier workloads, half noted increased migration and turnover and over one‐third encountered severe recruitment and retention challenges [8]. In Taiwan, prolonged postpandemic strain has accelerated nurse attrition—turnover rose from 11.12% in 2019 to 12.61% in 2023, and vacancy rates nearly doubled from 4.52% to 9.05% [9]. Notably, national statistics indicate a gradual ageing of the nursing workforce, characterised by a relative increase in nurses aged over 40 years and structural shifts across younger and midcareer age groups, rather than a uniform pattern of attrition within any single age cohort [10]. These demographic changes underscore potential heterogeneity in the experiences of nurses in early and midcareer stages, whose roles and expectations may vary substantially across age and tenure, thereby warranting further empirical examination.

Organisational commitment plays a central role in maintaining both workforce stability and the quality of care delivery. It embodies an individual’s sense of belonging, identification with institutional goals and readiness to invest effort in the organisation. Conceptually, organisational commitment encompasses three core aspects—value, effort and retention—representing the alignment of personal beliefs and behaviours with organisational expectations [11]. The quality of interpersonal relationships, workplace climate and balance between professional and personal life all shape the degree of an individual’s commitment [12]. Research indicates that most nurses are willing to exceed role expectations to fulfil their duties [13], reflecting a generally moderate‐to‐high level of professional loyalty [6]. Nonetheless, factors such as age, marital status, education, clinical unit and tenure may influence the strength and consistency of this commitment.

Work perception captures how employees emotionally interpret their professional environment, assigning personal meaning—positive or negative—to daily experiences. Positive perceptions, such as work excitement, support mental well‐being and work engagement, whereas negative perceptions, such as work frustration, foster fatigue and turnover intentions [14, 15]. Opportunities for learning and advancement foster positive perceptions, whereas chronic staffing shortages and inflexible schedules are major sources of frustration. Peer and supervisory support, promotion systems, departmental culture, health status and workload are also important influencing factors [16]. Taken together, these patterns indicate that nurses’ work perceptions reflect an ongoing interplay between demanding aspects of work and the availability of supportive and motivating conditions in the workplace. The job demands–resources (JD‐R) model offers a comprehensive framework for explaining the relationship between nurses’ work excitement, work frustration and organisational commitment.

The core premise of the JD‐R model [17] posits that job demands and job resources constitute two fundamental categories of work characteristics that shape employees’ work‐related outcomes through distinct motivational and strain‐related emotional processes. Job resources operate through a motivational process that enhances employees’ work motivation and organisational commitment [18]. When employees perceive sufficient and stable resources in their work environment, the motivational process is activated, increasing the likelihood that their emotional experiences manifest as work excitement, a positive and motivational work‐related affective state characterised by enthusiasm, vitality and positive engagement with one’s work [18, 19]. This mechanism has also been empirically demonstrated among nurses working in high‐demand clinical settings [20]. Such emotional experiences facilitate greater work engagement and a stronger sense of professional value, foster identification with organisational goals and ultimately strengthen employees’ willingness to remain with and invest effort in their organisation, thereby enhancing organisational commitment [21].

In contrast, when employees are exposed to persistently high job demands in the absence of adequate compensatory resources, job demands activate a strain process characterised by the accumulation of resource depletion, health complaints and negative emotional reactions [17, 18]. Within this process, employees’ subjective emotional responses to excessive job demands may be expressed as work frustration, reflecting the psychological burden experienced under conditions of high pressure and insufficient resources [20]. When such negative emotional experiences persist over time, they are likely to erode employees’ affective attachment to the organisation and weaken their intention to sustain organisational commitment. Building on this perspective, individuals continuously engage in cognitive and emotional processing through which they construct subjective interpretations of their work environment. As a result, work perception extends beyond a mere evaluation of working conditions and represents a lived experience that shapes employees’ work engagement and psychological resilience. In this sense, work excitement and work frustration can be understood as affective manifestations of employees’ work perception within the JD‐R framework.

Taken together, the persistent nursing shortage and the sustained high‐pressure work environment in the postpandemic era have rendered nurses’ organisational commitment a critical issue for healthcare system stability and the quality of care. As the nursing workforce continues to age, midcareer nurses now account for an increasing proportion of the workforce. This group is characterised by substantial heterogeneity in accumulated work experience, role expectations and psychological demands, indicating that midcareer nursing represents a distinct career stage warranting focused investigation. Drawing on the JD‐R model and existing evidence, work excitement and work frustration can be conceptualised as key affective outcomes within nurses’ work processes that, in turn, shape organisational commitment. However, prior research has rarely centred on midcareer nurses or systematically examined the relationship between affective work perceptions and organisational commitment. This gap limits understanding of the mechanisms underlying retention and organisational stability among midcareer nurses.

## 2. Aim

In response to the impact of midcareer nurse turnover on healthcare capacity and quality of care in Taiwan, this study focused on midcareer nurses and aimed to (1) examine differences in organisational commitment among midcareer nurses across demographic and work‐related characteristics and (2) investigate the relationships between work excitement and work frustration and organisational commitment among midcareer nurses to identify key predictors of organisational commitment within this career stage.

## 3. Materials and Methods

### 3.1. Study Design

This study employed a cross‐sectional design using convenience sampling. Registered nurses working in healthcare facilities across northern Taiwan were invited to participate through public recruitment posts disseminated via online professional nursing social media platforms. Data collection was conducted between July and December 2024. The study followed the Strengthening the Reporting of Observational Studies in Epidemiology (STROBE) statement, as recommended by the EQUATOR Network [22].

### 3.2. Participants and Settings

Participants were eligible for inclusion if they met the following criteria: (1) being aged 18 years or above; (2) proficiency in Mandarin, Taiwanese or Hakka; (3) full‐time employment at or above the regional hospital level; and (4) possession of a valid nursing licence and at least 3 months of clinical work experience. Consistent with the study aim, midcareer nurses were conceptualised as a career‐stage group rather than defined by restrictive age or tenure cutoffs. Therefore, age and years of clinical experience were not used as exclusion criteria but were examined analytically to capture variations across career stages, with particular attention to issues relevant to the midcareer phase.

Sample adequacy was determined using G∗Power Version 3.1, referencing a study from Myanmar (*N* = 234) [23]. The parameters were set for an F‐test of linear multiple regression (fixed model, deviation of *R* ^2^ from zero), with an assumed effect size of *f* ^2^ = 0.13, *α* = 0.05, statistical power of 0.90, and 10 predictors. With an actual sample of 240 nurses (df1 = 10, df2 = 229), the achieved power was approximately 0.98, indicating sufficient statistical sensitivity for the study design.

### 3.3. Data Collection

Recruitment materials, including an electronic information sheet detailing the study purpose, procedures and ethical considerations, together with a QR code linking to the online survey platform, were disseminated through professional online nursing groups. Participation was entirely voluntary, and no incentives were offered. After reviewing the study information, eligible nurses who agreed to participate accessed the online questionnaire system and provided electronic informed consent prior to completing the survey. All responses were submitted anonymously, with no personally identifiable information collected. Completed questionnaires were automatically stored in a secure database accessible only to the research team. Data were subsequently coded and anonymised for analysis to ensure confidentiality and data protection.

### 3.4. Sociodemographic Questionnaire

The sociodemographic questionnaire obtained data on age, relationship status, education level, hospital level, tenure, position, shift type and workload.

### 3.5. Work Perception

Work perception was assessed using the validated Chinese versions of the Work Excitement Scale (WES) and Work Frustration Scale (WFS), originally developed by Erbin‐Roesemann and Simms and subsequently revised by Chang et al. [14]. Each instrument comprises 17 items measured on a four‐point Likert scale ranging from 1 (‘*not at all*’) to 4 (‘*very much*’). Higher scores reflect greater levels of work excitement or frustration. The WES evaluates three dimensions: utilisation and development of professional skills (7 items), acquisition of new knowledge (6 items) and the level of challenge and diversity in nursing work (4 items). The WFS measures three domains of occupational stress: interpersonal relationships (8 items), resource use (5 items) and work management (4 items). Both scales demonstrated satisfactory psychometric properties in previous studies, with factor loadings above 0.6 and Cronbach’s *α* values exceeding 0.90 for the total scales and subscales. Chang et al. [16] used these scales to study the work experiences of 165 senior nurses, with Cronbach’s *α* values of the WES ranging from 0.80 to 0.87 and of the WFS ranging from 0.84 to 0.94. In the present study, Cronbach’s *α* coefficient was 0.88. In the present study, internal consistency reliability was acceptable to excellent. Cronbach’s *α* coefficient for the WES was 0.90, with subscale *α* values of 0.86, 0.84, and 0.83, respectively. Cronbach’s *α* coefficient for the WFS was 0.94, with corresponding subscale *α* values of 0.92, 0.91, and 0.83.

### 3.6. Organisational Commitment

Organisational commitment was evaluated using the instrument developed by Chih et al. [11]. The scale contains 10 items divided into three subdimensions: retention commitment (4 items), value commitment (4 items) and effort commitment (2 items). Each item is rated on a seven‐point Likert scale from 1 (‘*strongly disagree*’) to 7 (‘*strongly agree*’), with higher scores representing stronger organisational commitment. The reliability and validity of this tool have been confirmed among healthcare professionals in Taiwan. Psychometric evaluation by Chih et al. demonstrated satisfactory internal consistency, with Cronbach’s *α* coefficients ranging from 0.77 to 0.93 for the total scale and its subscales. Confirmatory factor analysis further supported the construct validity of the scale, yielding a comparative fit index (CFI) of 0.96 (*p* < 0.001), indicating excellent model fit and suitability for assessing organisational commitment in Taiwanese nursing contexts. For this study, the overall internal consistency of the scale was high, with a Cronbach’s *α* coefficient of 0.92. Reliability estimates for the three subdimensions were 0.81, 0.91, and 0.66, respectively.

### 3.7. Statistical Analysis

Data analysis was conducted using IBM SPSS Statistics Version 22.0. Descriptive statistics, including frequencies, percentages, means and standard deviations, were used to summarise participants’ demographic and work‐related characteristics. Continuous variables were expressed as means and standard deviations, while categorical variables were presented as frequencies and proportions.

Independent sample *t*‐tests and one‐way analyses of variance (ANOVA) were performed to identify differences in organisational commitment across demographic subgroups. Age and tenure were categorised for comparative purposes: age was divided into ≤ 30, 31–40 and > 40 years, and work experience into < 3, 3–8, 9–15 and > 15 years. These groupings were established based on the sample distribution and key transitional stages in nursing career progression, consistent with prior literature and clinical relevance.

Categorical variables were dummy‐coded for inclusion in regression analyses. Multiple linear regression was then applied to determine the predictors of organisational commitment. A two‐tailed *p* value of < 0.05 was considered statistically significant. Ninety‐five percent confidence intervals (CIs) were reported to indicate the precision of all estimates and regression coefficients.

### 3.8. Ethical Considerations

Ethical approval for this study was granted by the Institutional Review Board of Cheng Hsin General Hospital, northern Taiwan (Approval No. (1105)113‐35; approved on 8 July 2024, valid until 7 July 2025). Permission to use the study instruments was obtained from the respective authors prior to data collection. Participation was entirely voluntary, and completion of the online questionnaire was taken as informed consent. All responses were anonymised, coded and delinked from personal identifiers to ensure confidentiality. The study strictly adhered to the ethical standards for human subject research and complied with institutional and national data protection guidelines.

## 4. Results

### 4.1. Participant Characteristics

Table 1 summarises the demographic characteristics of the participants. A total of 240 female nurses were included in the analysis, with a mean age of 32.54 years (SD = 8.46) and an average work experience of 10.36 years (SD = 8.08). The majority of the participants were employed at regional hospitals (63.7%) and had 3–8 years of work experience (32.1%).

**TABLE 1:** Demographic characteristics of participants (*N* = 240).

Variable	*n* (%)	Mean ± SD
Age (years)		32.54 ± 8.46
① ≤ 30	131 (54.6)	
② 31–40	62 (25.8)	
③ > 40	47 (19.6)	
Relationship status		
Unmarried/divorced/widowed	158 (65.8)	
Married	82 (34.2)	
Education		
Junior college	63 (26.3)	
University and above	177 (73.8)	
Hospital level		
① District hospital	19 (7.9)	
② Regional hospital	153 (63.7)	
③ Medical centre	68 (28.3)	
Tenure (years)		10.36 ± 8.08
① < 3	51 (21.3)	
② 3–8	77 (32.1)	
③ 9–15	58 (24.2)	
④ > 15	54 (22.5)	
Position		
Nonmanagerial position	189 (78.8)	
Managerial position	51 (21.3)	
Shift type		
Fixed	55 (22.9)	
Rotating	185 (77.1)	
Workload level		
Low workload	95 (39.6)	
Heavy workload	145 (60.4)	

*Note: M*: mean.

Abbreviation: SD = standard deviation.

### 4.2. Organisational Commitment and Work Perception Status

As shown in Table 2, significant differences in organisational commitment were observed across age, tenure, managerial role, shift type and workload across all dimensions (all *p* < 0.05). No significant differences were observed in relationship status and hospital level for effort commitment and in hospital level for retention commitment. Additionally, education level was not significantly associated with differences in value commitment. Table 3 presents group comparisons of work perception. Significant differences in work excitement were found across relationship status, shift type and workload level (*p* < 0.05). In contrast, work frustration did not demonstrate significant between‐group differences across demographic variables.

**TABLE 2:** Differences in organisational commitment and its dimensions (*N* = 240).

Demographic variables	Total	*p*/Tukey	Effort	*p*/Tukey	Retention	*p*/Tukey	Value	*p*/Tukey
	*M* ± SD		*M* ± SD		*M* ± SD		*M* ± SD	
Age (years)		0 < 0.001		0.012		< 0.001		< 0.001
① ≤ 30	40.36 ± 9.75	② = ③ > ①	9.52 ± 2.19	③ > ①	16.37 ± 4.14	③ > ①	14.47 ± 4.70	② = ③ > ①
② 31–40	44.52 ± 9.84		10.05 ± 2.09		17.58 ± 4.24		16.89 ± 4.63	
③ > 40	48.60 ± 11.04		10.60 ± 2.29		19.47 ± 4.60		18.53 ± 5.12	
Relationship status		0.025		0.247		0.018		0.032
Unmarried	41.88 ± 9.61		9.75 ± 2.17		16.77 ± 4.06		15.36 ± 4.67	
Married	45.29 ± 11.79		10.10 ± 2.30		18.28 ± 4.88		16.91 ± 5.53	
Education level		0.186		0.712		0.030		0.482
Junior college	41.54 ± 12.18		9.78 ± 2.36		16.25 ± 5.12		15.51 ± 5.58	
University and above	43.58 ± 9.83		9.90 ± 2.17		17.66 ± 4.07		16.03 ± 4.82	
Hospital level		0.132		0.982		0.176		0.038
① District hospital	42.63 ± 7.58		9.89 ± 1.88		16.74 ± 3.49		16.00 ± 3.51	① = ② > ③
② Regional hospital	44.03 ± 10.70		9.88 ± 2.13		17.69 ± 4.53		16.46 ± 5.05	
③ Medical centre	40.96 ± 10.59		9.82 ± 2.50		16.54 ± 4.28		14.59 ± 5.13	
Tenure (years)		< 0.001		0.001		< 0.001		< 0.001
① < 3	41.63 ± 9.86	③ = ④ > ② = ①	9.92 ± 2.18	③ = ④ > ②	16.43 ± 4.37	③ = ④ > ② = ①	15.27 ± 4.48	③ = ④ > ② = ①
② 3–8	39.16 ± 9.38		9.09 ± 2.07		16.06 ± 4.04		14.00 ± 4.74	
③ 9–15	45.07 ± 9.63		10.17 ± 2.19		18.00 ± 4.00		16.90 ± 4.59	
④ > 15	47.76 ± 11.37		10.59 ± 2.20		19.07 ± 4.70		18.09 ± 5.32	
Managerial role		< 0.001		0.019		< 0.001		< 0.001
Nonmanagerial	41.68 ± 10.30		9.69 ± 2.18		16.77 ± 4.38		15.22 ± 4.96	
Managerial	48.10 ± 9.79		10.51 ± 2.25		19.22 ± 3.99		18.37 ± 4.49	
Shift type		0.008		0.010		0.042		0.010
Fixed	46.31 ± 9.05		10.55 ± 2.08		18.35 ± 3.94		17.42 ± 4.21	
Rotating	42.08 ± 10.74		9.66 ± 2.22		16.97 ± 4.49		15.77 ± 5.17	
Workload level		< 0.001		0.004		< 0.001		0.007
Low workload	45.84 ± 8.91		10.38 ± 2.06		18.49 ± 3.65		16.97 ± 4.47	
Heavy workload	41.21 ± 11.08		9.53 ± 2.26		16.50 ± 4.68		15.19 ± 5.25	

*Note: M*: mean.

Abbreviation: SD = standard deviation.

**TABLE 3:** Differences in work excitement and work frustration (*N* = 240).

Demographic variables	Work excitement	*p*	Work frustration	*p*
	*M* ± SD		*M* ± SD	
Age (years)		0.219		0.313
① ≤ 30	42.56 ± 7.42		49.43 ± 10.77	
② 31–40	44.31 ± 8.00		46.92 ± 11.44	
③ > 40	44.28 ± 7.84		47.77 ± 12.02	
Relationship status		0.034		0.070
Unmarried	42.59 ± 7.70		49.40 ± 10.38	
Married	44.80 ± 7.45		46.63 ± 12.50	
Education level		0.021		0.298
Junior college	41.43 ± 7.34		47.19 ± 11.37	
University and above	44.03 ± 7.69		48.90 ± 11.14	
Hospital level		0.203		0.589
① District hospital	42.32 ± 6.71		47.68 ± 10.13	
② Regional hospital	42.86 ± 7.45		48.03 ± 10.79	
③ Medical centre	44.74 ± 8.31		49.63 ± 12.40	
Tenure (years)		0.231		0.299
① < 3	42.04 ± 6.77		50.06 ± 8.72	
② 3–8	42.70 ± 7.44		49.16 ± 11.95	
③ 9–15	44.72 ± 8.41		46.21 ± 11.77	
④ > 15	44.02 ± 7.87		48.35 ± 11.47	
Managerial role		0.099		0.430
Nonmanagerial	42.92 ± 7.56		48.75 ± 11.18	
Managerial	44.92 ± 7.95		47.35 ± 11.33	
Shift type		0.001		0.774
Fixed	46.22 ± 6.51		48.84 ± 10.61	
Rotating	42.49 ± 7.80		48.38 ± 11.40	
Workload level		0.012		0.390
Low workload	44.88 ± 7.28		47.68 ± 11.39	
Heavy workload	42.34 ± 7.78		48.96 ± 11.09	

*Note: M*: mean.

Abbreviation: SD = standard deviation.

### 4.3. Multiple Regression Analysis of Organisational Commitment in Front‐Line Nurses

Table 4 shows the results of multiple regression analyses predicting organisational commitment. After controlling for relevant demographic variables, workload and work excitement emerged as significant predictors across all dimensions of organisational commitment (*p* < 0.05). In addition, nurses aged over 40 years showed higher value commitment, while those with 3–8 years of tenure demonstrated lower effort commitment compared to their reference groups. Heavy workload was negatively associated with effort, retention and value commitment, whereas higher work excitement was positively associated with all three dimensions. Work frustration showed a modest but significant negative association with retention and value commitment. All regression models were statistically significant (effort commitment: *F* = 8.27; retention commitment: *F* = 9.17; value commitment: *F* = 7.78; all *p* < 0.001), explaining 29%–34% of the variance in organisational commitment.

**TABLE 4:** Multiple linear regression analyses predicting the three dimensions of organisational commitment (*N* = 240).

Predictor variables	Effort		95% CI Lower/upper	Retention		95% CI Lower/upper	Value		95% CI Lower/upper
	*β*	*t*/*F*		*β*	*t*/*F*		*β*	*t*/*F*	
(Constant)		8.27^∗∗∗^	—		9.17^∗∗∗^	—		7.78^∗∗∗^	—
Age (ref: ≤ 30 years)									
31–40 years	−0.05	−0.54	−1.27/0.72	−0.04	−0.41	−2.37/1.54	0.18	1.80	−0.18/4.24
> 40 years	−0.04	−0.35	−1.64/1.14	0.11	0.95	−1.39/4.03	0.27	2.14^∗^	0.27/6.44
Relationship status (ref: unmarried)									
Married	—	—	—	−0.06	−0.92	−1.86/0.66	−0.13	−1.90	−2.83/0.05
Education level (ref: junior college)									
University	−0.08	−1.37	−1.00/0.17	0.05	0.91	−0.61/1.67	—	—	—
Hospital level (ref: district hospital)									
Regional hospital	—	—	—	—	—	—	0.06	0.53	−1.60/2.79
Medical centre	—	—	—	—	—	—	−0.12	−1.16	−3.60/0.93
Tenure (ref: < 3 years)									
3–8 years	−0.20	−2.73^∗∗^	−1.65/−0.26	−0.07	−0.97	−2.00/0.68	−0.14	−1.90	−3.04/0.05
9–15 years	−0.01	−0.02	−1.09/1.06	0.06	0.63	−1.43/2.79	−0.07	−0.66	−3.26/1.62
> 15 years	0.11	0.82	−0.82/2.01	0.12	0.92	−1.47/4.09	−0.01	−0.00	−3.18/3.15
Position (ref: nonmanagerial)									
Managerial	0.06	0.94	−0.36/1.02	0.11	1.83	−0.08/2.58	0.08	1.27	−0.56/2.62
Shift type (ref: fixed)									
Rotating	−0.01	−0.07	−0.68/0.63	0.06	1.07	−0.57/1.97	0.01	0.19	−1.33/1.63
Workload level (ref: low workload)									
Heavy workload	−0.14	−2.50^∗^	−1.18/−0.14	−0.17	−2.98^∗∗^	−2.52/−0.51	−0.15	−2.70^∗∗^	−2.68/−0.42
Work excitement	0.42	7.15^∗∗∗^	0.08/0.15	0.41	7.05^∗∗∗^	0.16/0.29	0.32	5.62^∗∗∗^	0.13/0.28
Work frustration	−0.05	−1.03	−0.03/0.01	−0.15	−2.60^∗∗^	−0.10/−0.01	−0.23	−4.15^∗∗∗^	−0.15/−0.05
*R* ^2^	0.29			0.33			0.34		

*Note: β* = standardised regression coefficient. Separate regression models were fitted for each dimension of organisational commitment, with demographic and work‐related characteristics entered as control variables depending on model specification.

Abbreviation: CI = confidence interval.

^∗^ *p* < 0.05.

^∗∗^ *p* < 0.01.

^∗∗∗^ *p* < 0.001.

## 5. Discussion

This study revealed that several demographic characteristics meaningfully shaped Taiwanese nurses’ organisational commitment. Those who were older, had longer professional tenure, were married, occupied senior positions or worked in more stable and less demanding schedules reported stronger commitment to their organisations. Among these variables, age and tenure appeared to be particularly influential. This aligns with earlier evidence [24, 25] suggesting that accumulated experience and occupational continuity foster professional identification and emotional connection with the workplace, which in turn reinforce retention, coping capacity and resilience [26, 27]. Moreover, enjoyment derived from work emerged as an independent predictor of commitment, even after accounting for demographic covariates. Intrinsic work satisfaction strengthens motivation, encouraging greater engagement and reinforcing both belonging and loyalty to the employing institution. Such positive affective experiences promote meaning‐making and professional confidence, which together consolidate long‐term organisational commitment [15, 28].

Findings further demonstrated that unfavourable work perceptions—particularly frustration and disillusionment—negatively affected nurses’ organisational commitment and were strong predictors of attrition risk. This tendency has intensified alongside the digital transformation of healthcare, where rapid technological adoption has altered workflow dynamics. Although automation has eased certain repetitive tasks, approximately one‐third of nurses report marked frustration and compassion fatigue, largely owing to limited familiarity with digital tools and inadequate training opportunities [29, 30]. Contributing organisational factors, such as resource constraints, staffing imbalances, role ambiguity and administrative overload, undermine both efficiency and perceived control, heightening job insecurity [15, 31]. Sustained exposure to such conditions fosters emotional exhaustion and depersonalisation [32], eroding organisational loyalty and reinforcing intentions to leave [33, 34].

Irregular shifts, high work intensity and unpredictable schedules hinder nurses’ ability to sustain equilibrium between work and personal life. Such disruption in role balance frequently results in inter‐role conflict [35]. Work–family incongruence induces psychological strain and emotional fatigue, manifesting as alienation, anxiety and depressive affect, which collectively compromise well‐being and weaken organisational attachment [36, 37].

The present study provides empirical evidence showing that nurses in the midcareer stage—approximately three to 8 years of experience—demonstrated lower levels of organisational commitment compared with both less‐experienced and more senior nurses. From a mental health perspective, this pattern aligns with the processes described in the concept of a midlife crisis, which involves heightened role strain, career stagnation and the reevaluation of occupational identity [38], and may also reflect a relatively low point in the U‐shaped trajectory of subjective well‐being across the life course [39]. A growing body of empirical evidence indicates that nurses in the midcareer stage, by virtue of their accumulated clinical competence and experience, assume higher‐level responsibilities requiring rapid and responsive patient care, while simultaneously facing pressures associated with difficulty in fully meeting organisational expectations [3]. In addition, persistent work‐related stress and constrained career development opportunities [27], performance‐oriented job demands and expectations [1], as well as observable health deterioration and emotional exhaustion [40, 41], may collectively undermine intrinsic work motivation and further reduce affective organisational commitment. Therefore, the observed ‘midcareer dip’ in organisational commitment may be viewed as an indicator of a psychologically vulnerable period in midcareer nurses’ professional trajectories, which is linked to lower levels of organisational loyalty and is also consistent with organisational behavioural research highlighting role strain and performance‐oriented work demands among midcareer professionals.

This observation corresponds with findings from the International Council of Nurses, which highlighted that the average nursing tenure in Taiwan is around 7.67 years, notably shorter than that of nurses in many Western nations. Earlier research has similarly reported that the anticipated professional lifespan of Taiwanese nurses averages about 8 years [42]. According to human capital theory, individuals accumulate professional knowledge, technical competence and practical insight over time, thereby enhancing their value to the organisation [43]. In theory, this accumulation should lead to proportional improvements in remuneration, benefits and career advancement, fostering stronger long‐term commitment [24]. Nevertheless, the current findings contradict this assumption. In the context of persistent workforce shortages and escalating workloads, extended tenure may no longer be associated with greater fulfilment but instead mark the onset of professional fatigue and disillusionment.

### 5.1. Challenges Faced by Midcareer Nurses

In the Taiwanese healthcare context, shift work functions not only as a scheduling arrangement but also as a structural constraint shaping nurses’ professional and personal lives. Among midcareer nurses, who often face heightened professional expectations alongside increasing family responsibilities, rotating and irregular shift patterns limit opportunities for physical and psychological recovery. This rigidity undermines work–life integration and intensifies emotional exhaustion and declining organisational commitment [44, 45].

Nurses following this career trajectory typically complete one to 3 years of foundational clinical training before progressing to advanced roles focused on administration and quality improvement (Taiwan Nurses Association). Those with three to 8 years of experience represent a pivotal cohort, combining practical expertise with professional maturity. However, this career stage also coincides with escalating clinical pressures and organisational expectations. Many encounter widening disparities between professional ideals and workplace realities, characterised by staff shortages, patient complexity, extended overtime and insufficient institutional support [46, 47].

Despite growing expertise, midcareer nurses frequently report limited avenues for advancement [48]. Although experience and competence are expected to correspond with improved status and remuneration [49], opaque promotion systems, a lack of structured career frameworks and minimal mentoring opportunities hinder progression. Consequently, capable nurses often perceive their contributions as undervalued, fuelling frustration, inequity and disillusionment that undermine job satisfaction and organisational commitment [34, 50].

This group also faces pronounced breaches in the psychological contract with their employers. Psychological contract theory posits that mutual expectations and implicit obligations between employees and organisations form the basis of reciprocal trust [51]. For many nurses, early professional enthusiasm gives way to perceived organisational indifference, particularly in settings with generational or tenure gaps that impede collaboration, continuity of care and knowledge transfer [52, 53]. Current workforce strategies in Taiwan largely prioritise short‐term staffing solutions over sustainable professional development, neglecting long‐term retention, role diversification and career advancement opportunities [8]. Addressing these systemic gaps requires transparent promotion pathways and targeted support for lifelong career progression.

International models offer valuable insights. In Australia, empowering experienced nurses through expanded roles and strategic engagement has improved retention and workplace satisfaction [54]. The South Australian job‐enrichment initiative demonstrated that diversified responsibilities in education, research and quality improvement fostered achievement and professional recognition, substantially reducing turnover [55]. Similarly, Sweden’s advanced practice mentoring programme utilised senior nurses as educators, cultivating a ‘second career curve’ that enhanced retention and intergenerational knowledge exchange [56]. The Queensland Health Framework for Lifelong Learning established structured career ladders, mentoring systems and flexible work arrangements to strengthen nurses’ professional identity and long‐term commitment [57]. These initiatives underscore that nursing expertise constitutes essential organisational capital. Tailored career development systems aligned with local contexts foster professional growth, emotional attachment and retention [7, 58, 59].

### 5.2. Local Adaptation Challenges and Cultural Considerations in Taiwan

Although international experiences provide valuable reference models, given that the majority of Taiwan’s nursing workforce is concentrated in hospital‐based care settings, characterised by relatively rigid staffing regulations and working time arrangements, limited nursing reimbursement and clinical performance evaluation systems that prioritise short‐term outcomes [60–62], the time and space available for nurses’ nonclinical professional development are severely constrained. Consequently, the direct application of international models continues to face considerable challenges in Taiwan.

Internationally, shift work is increasingly recognised as an organisational responsibility rather than an individual coping issue, prompting healthcare systems to adopt more flexible scheduling models. In this context, a self‐scheduling system was preliminarily implemented at Changhua Christian Hospital in Taiwan in early 2025, improving flexibility and transparency in nurses’ working hours [63]. However, within East Asian cultural contexts, nursing is often framed as a virtue‐based profession emphasising dedication, and together with entrenched workplace norms and concerns regarding the fairness of self‐scheduling, these factors continue to hinder broader implementation. Addressing these challenges requires organisational cultural change and policy‐level interventions focused on sustainable workforce planning and scheduling strategies sensitive to nurses’ age and career stage.

Taiwan’s current nursing competency advancement framework continues to emphasise horizontal skill differentiation and salary progression [64], while vertical development pathways in education, research and leadership remain underdeveloped. Midcareer nurses are frequently placed in a challenging position in which they are expected to contribute professionally and provide educational guidance yet receive limited formal recognition for these roles. Although the introduction of the advanced practice nurse role in Taiwan in 2000 represented a significant step forward [65], teaching responsibilities are often added without corresponding workload adjustments, making it difficult to achieve effective educational outcomes under unchanged clinical demands. Notably, in 2024, Taiwan established a clinical mentorship system similar to that implemented in Sweden, which clearly differentiates the clinical and educational roles of mid‐ and senior‐level nurses and helps reduce their clinical workload [66]. Nevertheless, given the substantial and persistent nursing workforce shortages in Taiwan, whether nurses serving as ‘clinical educators’ can genuinely be excluded from frontline staffing and devote themselves fully to the clinical teaching of junior nurses remains an issue that requires long‐term observation.

Unlike the competency‐mix–oriented nursing workforce configurations commonly adopted internationally, Taiwan’s current clinical workforce utilisation and organisational culture constrain the flexibility of mid‐ and senior‐career nurses to explore diversified career development pathways. In many Western countries, nursing teams are organised based on clearly defined educational qualifications, competencies and role delineations, integrating nurses of different levels and specialisations into care units. This approach supports role differentiation and professional deepening and enables some mid‐ and senior‐career nurses to transition into nonfull‐time clinical roles in education, research or quality management. In contrast, Taiwan’s nursing culture continues to place clinical practice at the core of professional value and workforce deployment, whereby direct clinical care is regarded as the primary—and often essential—responsibility of mid‐ and senior‐career nurses. This workforce and cultural logic not only weakens the feasibility of role differentiation but also leaves nonclinical or hybrid career pathways lacking institutional support and organisational legitimacy. Within this context, when mid‐ and senior‐career nurses attempt to develop competencies in education, research or leadership, they are often required to assume additional responsibilities without a corresponding reduction in clinical workload, thereby exacerbating role conflict and occupational burnout. In the longer term, future strategies should move toward multifaceted career development models encompassing clinical, managerial, academic and research domains, in order to sustain midcareer engagement and enhance the quality of nursing care [67].

Moreover, managerial strategies should account for individual differences and psychological well‐being, emphasising the cultivation of a supportive and psychologically safe workplace. Optimising shift patterns and ensuring balanced workloads, together with efficient resource allocation, are critical measures for maintaining workforce stability. Such initiatives are essential not only for strengthening nurses’ organisational commitment but also for sustaining organisational resilience and effectiveness in the face of an evolving healthcare landscape.

### 5.3. Limitations and Suggestions

Several limitations should be acknowledged. First, this study employed convenience sampling and recruited nurses exclusively from northern Taiwan, which may limit the generalisability of the findings. In Taiwan, healthcare contexts vary across the northern, central, southern and eastern regions in terms of hospital scale, workforce distribution and resource availability, which may influence nurses’ work experiences and organisational commitment. Future studies are therefore encouraged to adopt larger‐scale, island‐wide sampling strategies, preferably using probability or randomised sampling methods, to validate and extend the present findings. Second, given that male nurses constitute only 4.5% of the Taiwanese nursing workforce, gender comparisons were not possible. As gender differences may influence occupational attitudes and commitment, future studies should include male participants to provide a more comprehensive perspective. Finally, the cross‐sectional design restricts inference regarding temporal or causal relationships. Longitudinal or mixed‐methods designs could better capture evolving patterns of organisational commitment and contextual influences over time.

## 6. Conclusions

This study examined the associations between demographic characteristics, work perceptions and the organisational commitment of nurses in the aftermath of a major infectious disease outbreak. The findings demonstrate that Taiwanese nurses’ commitment to their organisations is significantly shaped by both personal attributes and workplace experiences. Nurses who perceive their work environments positively tend to develop stronger identification with and attachment to their institutions, whereas unfavourable perceptions are linked to diminished commitment levels.

Notably, midcareer nurses exhibited comparatively lower levels of organisational commitment, highlighting a vulnerable stage within the nursing workforce that warrants particular attention. These findings underscore the importance of addressing workplace conditions and career development experiences in sustaining organisational commitment and workforce stability in Taiwan’s hospital‐based nursing system.

## Author Contributions

Conceptualisation, methodology, investigation, resources, supervision and writing–review and editing: Ching‐Wen Wei and Yen‐Chin Chen.

Data curation, formal analysis and writing–original draft: Ching‐Wen Wei.

Project administration and validation: Ching‐Wen Wei and Ching‐Wen Hu.

## Funding

This study was funded by the Taiwan Comprehensive University System (TCUS) (grant number 113TOP3‐2).

## Disclosure

All authors read and agreed before final submission. The funder had no role in study design, data collection, analysis, interpretation or manuscript preparation.

## Ethics Statement

This study was approved by the Institutional Review Board of Cheng Hsin General Hospital in northern Taiwan (Approval No. (1105)113‐35).

## Consent

The authors have nothing to report.

## Conflicts of Interest

The authors declare no conflicts of interest.

## Data Availability

The data and all materials used in this study are available from the corresponding author upon reasonable request.
